# Introduction to immunology and immune disorders

**DOI:** 10.1186/s13223-024-00932-5

**Published:** 2024-12-19

**Authors:** Jean S. Marshall, Julia E. M. Upton, Harissios Vliagoftis, Kyla J. Hildebrand, Adam Byrne, Wade Watson

**Affiliations:** 1 Department of Microbiology and Immunology, Dalhousie University, Halifax, NS Canada; 2 Division of Immunology and Allergy, Department of Pediatrics, Temerty School of Medicine, University of Toronto, The Hospital for Sick Children, Toronto, ON Canada; 3 Division of Pulmonary Medicine, Department of Medicine and Alberta Respiratory Centre, University of Alberta, Edmonton, AB Canada; 4 Division of Allergy and Immunology, Department of Pediatrics, Faculty of Medicine, University of British Columbia, Vancouver, BC Canada; 5 Division of Infectious Diseases, Immunology & Allergy, Children’s Hospital of Eastern Ontario, Ottawa, Canada; 6 Department of Pediatrics, Division of Allergy, Dalhousie University, IWK Health Centre, Halifax, NS Canada

## Abstract

The body has a collection of physical barriers to prevent infection, but once these are overcome, we rely on our immune systems to protect us against a wide variety of infections. The complex mechanisms through which this is achieved are grouped into two lines of defense called the “innate” and “adaptive” immune systems. The innate immune system provides a rapid and tailored response to infection or injury often associated with inflammation. Innate immunity also promotes the development of acquired immunity. Specific, long-lasting responses against a particular infection are dependent on acquired immunity, and these provide immune memory, such that if we encounter the same pathogen again, we are better protected. Many diseases are related to defects in immune function which can lead to either a weakened or overactive immune response. Autoimmune diseases (where the immune system attacks tissues or organs) and allergies (where the immune system responds inappropriately to substances in our environment) are just two examples of conditions resulting from immune function defects. Improved understanding of immune processes provides tremendous opportunities for enhanced immunization strategies and immune-based therapies. This article provides an overview of the main components and functions of the immune system, and also serves as a primer to help readers understand the immunopathological disorders discussed in the remainder of this supplement.

## Introduction

Immunology is a rapidly advancing field with many specialized areas of study. The complexity with which the immune system combats infection rivals the complexity of the numerous types of microbes that can cause disease. This article does not aim to provide an in-depth review of immunology. Its purpose is to provide medical students, residents, primary-care practitioners, and other healthcare professionals with an introduction to the main components and functions of the immune system in health and disease. This article will also provide background information that will aid in understanding the other articles in this supplement that focus on immunopathology.

## The immune system: innate and adaptive immunity

The immune system refers to a collection of cells, chemicals and processes that function to protect the body from infection and damage. At areas in contact with disease-causing organisms such as bacteria, viruses, fungi and parasites (collectively referred to as pathogens), the immune system provides ongoing active defense against infection. This activity is particularly important at body surfaces which interact with the external environment, such as the skin, airways, and gastrointestinal and reproductive tracts. In addition, the immune system protects us against cancer cells and toxins throughout the body. Beyond the structural and chemical barriers which protect us from infection, the immune system can be simplistically viewed as having two “lines of defense”, known as innate immunity and adaptive immunity. Innate immunity represents the first line of defense to an intruding pathogen. It is a defense mechanism that is used by the host immediately, or within hours, of encountering a pathogen or tissue damage. The innate immune response can be triggered by common chemical signals associated with multiple pathogens, such as structures found on bacterial or fungal cell walls. Adaptive immunity, on the other hand, is antigen-dependent and antigen-specific, which means it responds to a very precise chemical structure(s) (antigen[s]), and involves a lag time between exposure to the antigen and maximal response. The important hallmark of adaptive immunity is the capacity for memory, which enables the host to mount a more rapid and efficient immune response upon subsequent exposure to the antigen. This memory response allows the host to vigorously combat infection, and it is the basis for vaccination strategies. Innate and adaptive immunity are not mutually exclusive mechanisms of host defense, but rather are complementary and interactive, with defects in either system resulting in host vulnerability or inappropriate responses. Often, the innate immune response aids in directing a more rapid and effective acquired response to infection [1, 2].

### Innate immunity

Innate immunity can be viewed as comprising four types of defensive barriers: anatomic (skin and mucous membrane), physiologic (temperature, low pH and chemical mediators), endocytic and phagocytic, and inflammatory. Table 1 summarizes the non-specific host-defense mechanisms for each of these barriers. Cells and processes that are critical for effective innate immunity to pathogens that evade the initial anatomic barriers have been widely studied. Innate immunity to pathogens often relies on pattern recognition receptors (PRRs) which allow a limited range of immune cells to detect and respond rapidly to a wide range of pathogens that share common structures, known as pathogen associated molecular patterns (PAMPs). Examples of these include bacterial cell wall components, such as lipopolysaccharides (LPS), and double-stranded ribonucleic acid (RNA) produced during viral infection. In addition, the innate immune system responds to signals from dead and dying cells, allowing innate immunity to mobilize if the physical barriers that protect the body are damaged. These processes involve chemicals known as “alarmins” and damage-associated molecular patterns (DAMPs) produced in response to cell and tissue damage.

**Table 1:** Summary of non-specific host-defense mechanisms for barriers of innate immunity

Barrier	Mechanism
**Anatomic**	
Skin	• Mechanical barrier inhibits entry of microbes • Acidic environment (pH 3–5) inhibits growth of microbes
Mucous membrane	• Normal flora competes with other microbes and limit their growth through multiple mechanisms • Mucous entraps foreign microbes and limits attachment • Cilia and other responses (gut motility/coughing) propel microbes out of body
**Physiologic**	
Temperature	• Body temperature/fever response inhibits growth of some pathogens and enhances action of antimicrobial enzymes
Low pH	• Acidic pH of stomach kills most undigested microbes
Chemical mediators	• Lysozyme and other enzymes cleave bacterial cell wall • Interferon induces antiviral defenses • Complement system lyses microbes and facilitates phagocytosis
**Phagocytic/endocytic barriers**	
	• Immune cells internalize (endocytosis) and break down foreign macromolecules and toxins • Specialized cells (blood monocytes, neutrophils, tissue macrophages) are recruited to the site of infection and internalize (phagocytose), kill and digest pathogens
**Inflammatory barriers**	
	• Inflammatory mediators (cytokines and chemokines) recruit phagocytic cells to sites of infection and help initiate appropriate acquired immune responses • Inflammatory mediators enhance mucus production and turnover of epithelial cells at infected surfaces

Adapted from Turvey 2010 [1]

An important function of innate immunity is the rapid recruitment of immune cells to sites of infection and inflammation through the production of cytokines and chemokines (proteins involved in immune cell–cell communication and recruitment). Cytokine production during innate immunity mobilizes defense mechanisms throughout the body while also activating local cellular responses to infection or injury. Key inflammatory cytokines released during the early response to bacterial infection are tumour necrosis factor (TNF), interleukin 1 (IL-1) and interleukin 6 (IL-6). These cytokines are critical for aiding in cell recruitment and the local inflammation and increased mucus production which is essential for clearance of many pathogens. They also contribute to the development of fever. Dysregulated production of such inflammatory cytokines, so that they are produced excessively without infection, is often associated with inflammatory or autoimmune disease, making them important therapeutic targets. Chemokines, such as CXCL8 (IL-8) and CCL2, direct the movement of critical immune cells, such as neutrophils and monocytes, into tissues to combat infection. Cytokines and chemokines produced during the innate immune response also aid in the proper development of an effective adaptive immune response by enhancing the activity of antigen-presenting cells (APCs) (discussed later) and increasing the accumulation of cells within lymph nodes draining infection sites.

The complement system is a biochemical cascade that functions to identify and opsonize (coat) bacteria and other pathogens. It renders pathogens susceptible to phagocytosis, a process by which immune cells engulf microbes and remove cell debris, and kills some pathogens and infected cells directly. The phagocytic action of the innate immune response promotes clearance of dead cells or antibody complexes and removes foreign substances present in organs, tissues, blood, and lymph. It can also activate the adaptive immune response through the mobilization and activation of APCs [1, 3].

**Fig. 1:**
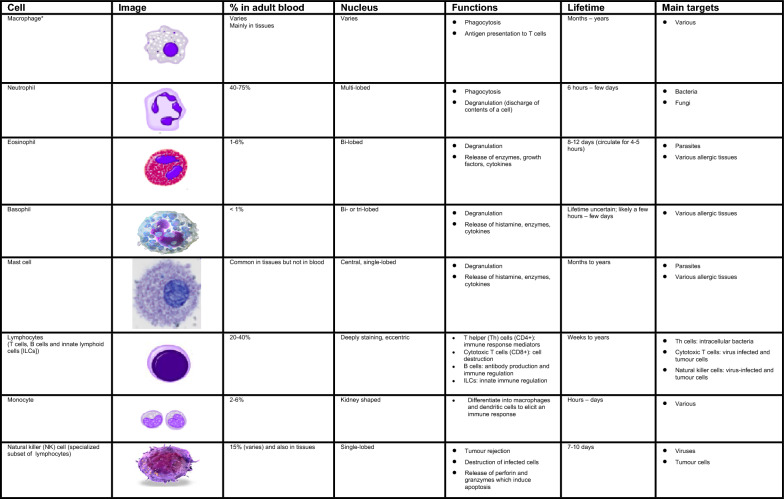
Characteristics and function of cells involved in innate immunity [1, 3, 4]. *Include alveolar macrophages (within pulmonary alveolus), histiocytes (connective tissue), Kupffer cells (liver), microglial cells (neural tissue), epithelioid cells (granulomas), osteoclasts (bone), mesangial cells (kidney)

Numerous cells are involved in the innate immune response such as phagocytes (macrophages and neutrophils), dendritic cells, mast cells, basophils, eosinophils, natural killer (NK) cells, and innate lymphoid cells. The main characteristics and functions of these cells are summarized in Fig. 1 [1, 3, 4].

Phagocytes are sub-divided into two main cell types: neutrophils and macrophages. Both cell types share a similar function: to engulf (phagocytose) microbes and kill them through multiple bactericidal pathways. In addition to their phagocytic properties, neutrophils contain granules and enzyme pathways that assist in the elimination of pathogenic microbes. Unlike neutrophils, which are short-lived cells mobilized rapidly to sites of infection, macrophages are long-lived cells that patrol the body and remove pathogens, dead cells, and debris. These cells not only play a role in phagocytosis, but are also involved in antigen presentation to T cells [1].

Dendritic cells also phagocytose and function as APCs, initiating the acquired immune response and acting as important linking cells between innate and adaptive immunity. Mast cells and basophils share many features with each other, and both are instrumental in the initiation of acute inflammatory responses, such as those seen in allergy and asthma. Mast cells also have important functions as immune “sentinel cells” and are early producers of cytokines in response to infection or injury. Unlike mast cells, which generally reside in the connective tissue surrounding blood vessels and are particularly common at mucosal surfaces, basophils generally reside in the circulation and are recruited to tissues following infection or during chronic allergic disease. Eosinophils are granulocytes that possess phagocytic properties and play an important role in the destruction of parasites that are often too large to be phagocytosed through toxic granule product release. Along with mast cells and basophils, they are also associated with allergy and asthma. NK cells play a major role in the rejection of tumours and the destruction of cells infected by viruses. Destruction of infected cells is achieved through the release of perforins and granzymes (proteins that cause lysis of target cells) from NK-cell granules which induce programmed cell death or apoptosis [4]. NK cells are also an important source of another cytokine, interferon-gamma (IFN-γ), which helps to mobilize APCs and promote the development of effective antiviral adaptive immunity. NK cells are part of a larger group of innate lymphoid cells (ILCs); the other members of this family play a more regulatory role. Depending on their type (i.e., ILC-1, ILC-2, ILC-3), they selectively produce cytokines such as IL-4, IFN-γ, and IL-17, respectively, that help to direct the appropriate early immune response to specific pathogens and contribute to immune regulation in that tissue but do not require specific antigen stimulation to do so.

### Adaptive immunity

The development of adaptive immunity is aided by the actions of the innate immune system. Adaptive immunity is critical when innate immunity is ineffective in fully eliminating infectious agents and for providing a “memory” enhanced response to repeated infection (e.g., to seasonal viruses like influenza or severe acute respiratory syndrome coronavirus 2 [SARS-CoV-2]). The primary functions of the adaptive immune response are the recognition of specific “non-self” antigens associated with pathogens or other threats, the generation or enhancement of immune effector pathways that can eliminate specific pathogens or pathogen-infected cells, and the development of an immunologic memory that can quickly eliminate a specific pathogen should subsequent infections occur. Adaptive immune responses are the basis for effective immunization against infectious diseases. The cells of the adaptive immune system include antigen-specific T cells, which are activated to proliferate through the action of APCs and have several roles in combatting infections and cancer, as well as B cells which differentiate into plasma cells to produce antibodies.

#### T cells and APCs

T cells derive from hematopoietic stem cells in bone marrow and, following migration, mature in the thymus. These cells express a series of unique antigen-binding receptors on their membrane, known as T-cell receptors (TCR). Each T cell expresses a single type of TCR, which binds to a particular unique chemical structure on an antigen. There are several thousand unique TCR on T cells in every individual. These T cells have the capacity to rapidly divide, proliferate, and differentiate into a T cell type(s) with specific roles in immunity. These roles include regulation of the immune system, promotion of antibody production by B cells and direct killing of virally infected or cancer cells. T cells divide and proliferate only if they receive the appropriate signals through the action of APCs (usually dendritic cells, but sometimes macrophages, B cells or structural cells) to recognize a specific antigen.

The surfaces of APCs express a group of proteins known as the major histocompatibility complex (MHC). MHC are classified as either class I (also termed human leukocyte antigen [HLA] A, B and C) which are found on all nucleated cells, or class II (also termed HLA-DP, -DQ and -DR) which are found only on certain cells of the immune system, including macrophages, dendritic cells, and B cells. Class I MHC molecules present endogenous (intracellular) peptides, such as viral peptides in virally infected cells, while class II molecules on APCs present exogenous (extracellular) peptides, such as those from many bacteria to T cells. These MHC proteins display fragments of antigens (peptides) when a cell is infected with an intracellular pathogen, such as a virus, or has phagocytosed foreign proteins or organisms [2, 3, 5].

**Fig. 2:**
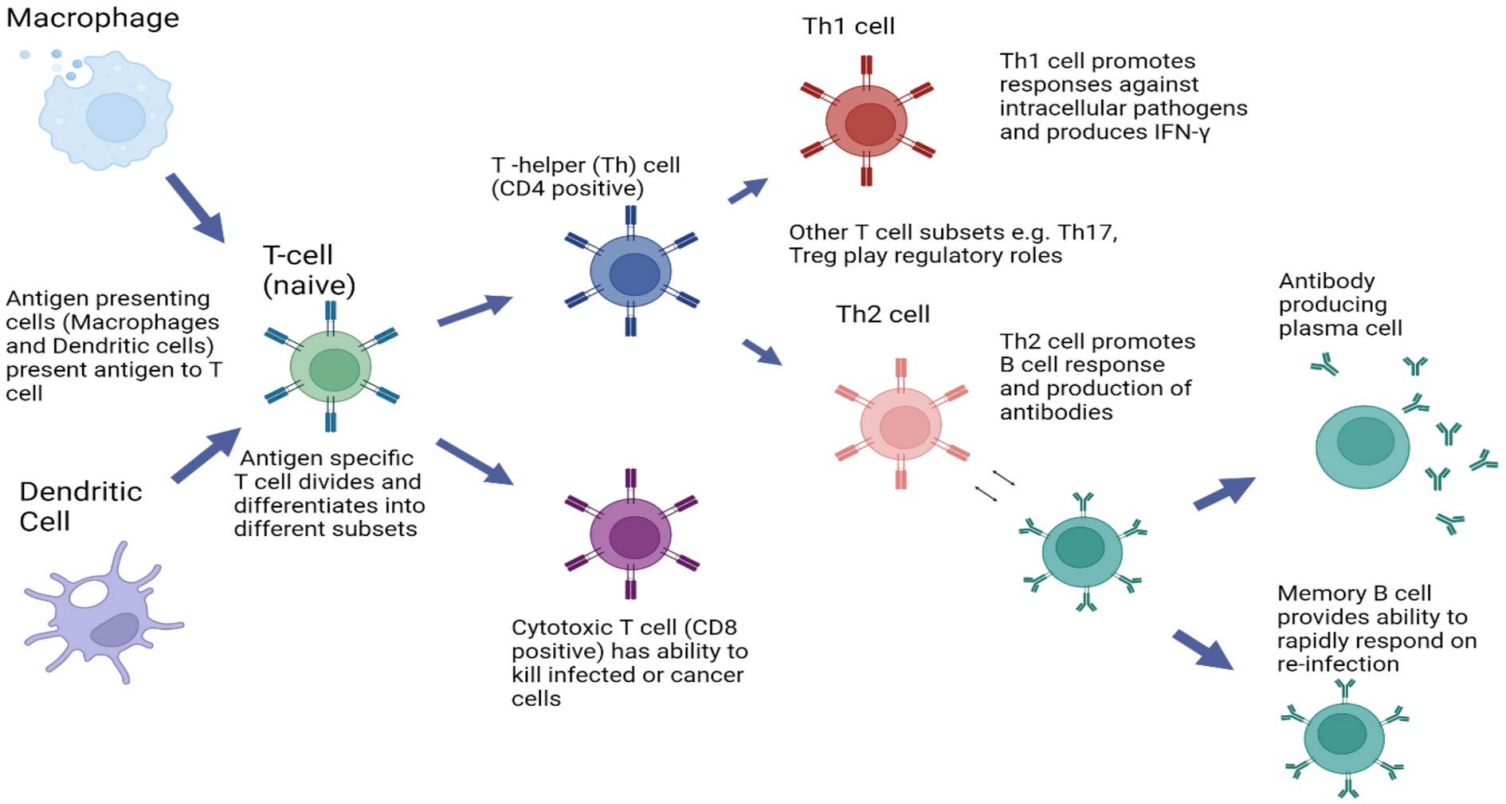
Fundamentals of the adaptive immune response. Figure created using Biorender

T cells have a wide range of unique TCRs which can bind to specific foreign peptides. During the development of the immune system, T cells that would react to antigens normally found in our body are largely eliminated, and other immune mechanisms reduce T-cell responses to items usually found in our environment, such as food. T cells are activated when they encounter an APC that has digested an antigen and is displaying the correct antigen fragments (peptides) bound to its MHC molecules to bind to that T cell’s specific TCR. The opportunities for the correct T cells to be in contact with an APC carrying the appropriate peptide MHC complex are increased by the circulation of T cells throughout the body (via the lymphatic system and blood stream) and their accumulation (together with APCs) in lymph nodes, a process that is enhanced by lymph node swelling initiated by the innate immune response. The MHC-antigen complex activates the TCR and the T cell secretes cytokines which further control the immune response. This antigen presentation process, in combination with signals provided by local cytokines, stimulates T cells to differentiate primarily into cytotoxic T cells (CD8 + cells) or T-helper (Th) cells (CD4 + cells) (see Fig. 2).

CD8 + cytotoxic T cells are primarily involved in the destruction of cells infected by foreign agents, such as viruses or intracellular bacteria, as well as the killing of some types of tumour cells. These cells are activated by the interaction of their TCR with peptide bound to MHC class I molecules, which can be found on most cells. Clonal expansion of cytotoxic T cells produces effector cells which release substances that induce apoptosis of target cells. Upon resolution of the infection, most effector cells die and are cleared by phagocytes. However, a few of these cells are retained as memory cells that can quickly differentiate into effector cells upon subsequent encounters with the same antigen or pathogen [3, 5].

CD4 + T helper (Th) cells play an important role in establishing and maximizing the immune response. These cells have no cytotoxic or phagocytic activity and cannot directly kill infected cells or clear pathogens. Instead, they promote the immune response by directing other cells to perform these tasks and regulate the type of immune response that develops. Th cells are activated through TCR recognition of antigen bound to class II MHC molecules. Once activated, Th cells release cytokines that influence the activity of many cell types, including the APCs that activate them. The responses of CD4 cells include the production of specialized cytokines such as IL-4, IL-17 and IFN-γ that can act to enhance other arms of the immune response, such as the production of antibodies by B cells, the killing activity of NK cells, and the ability of macrophages to take up and destroy bacteria.

Several types of Th cell responses can be generated, with Th1, Th2 and Th17 being the most frequent. The Th1 response is characterized by the production of IFN-γ which activates the bactericidal activities of macrophages and enhances antiviral immunity as well as immunity to other intracellular pathogens. Th1-derived cytokines also contribute to the differentiation of B cells to make opsonizing antibodies that enhance the efficiency of phagocytes. An inappropriate Th1 response is associated with certain autoimmune diseases.

The Th2 response is characterized by the release of cytokines (IL-4, 5, and 13) which promote the development of potent antibody responses to fight extracellular infections. These include immunoglobulin E (IgE) antibody-producing B cells, as well as the development and recruitment of mast cells and eosinophils that are essential for effective responses against many parasites. In addition, they enhance the production of certain forms of IgG that aid in combatting bacterial infection. As mentioned earlier, mast cells and eosinophils are instrumental in the initiation of acute inflammatory responses, such as those seen in allergy and asthma. IgE antibodies are also associated with allergic reactions (see Table 2). Therefore, an imbalance of Th2 cytokine production is associated with the development of atopic (allergic) conditions. In contrast, Th17 cells are characterized by the production of cytokines of the IL-17 family, and are associated with ongoing inflammatory responses, particularly in chronic infection and disease. This Th17 response is regulated by several other cytokines associated with inflammation, including IL-23 from T cells and other cells. Like cytotoxic T cells, most Th cells will die upon resolution of infection, with a few remaining as Th memory cells [3, 5].

**Table 2:** Major functions of human Ig antibodies [6]

Ig antibody	Function
IgM	• First immunoglobulin (Ig) produced in response to infection • Opsonizing (coats) infected cells or bacteria for destruction through complement fixation • Very efficient complement activator
IgG	• Main Ig during secondary immune response • Only antibody capable of crossing the placental barrier • Neutralization of toxins and viruses • Opsonizing (coating) antigen for destruction • Complement fixation
IgD	• Function unclear; appears to be involved in regulating the development of B cells and antibody responses
IgA	• Mucosal response; protects mucosal surfaces from toxins, viruses and bacteria through either direct neutralization or prevention of binding to mucosal surface • Found in large amounts in breast milk and mucosal secretions
IgE	• Associated with hypersensitivity and allergic reactions • Plays a role in immune responses to parasites

A further subset of the CD4 + T cells, known as “regulatory T cells” (T reg), also play a role in the immune response. T reg cells limit and suppress immune responses. They also help to prevent development of autoimmune diseases by reducing immune responses to self-antigens. T reg cells may also help in the resolution of normal immune responses, as pathogens or antigens are eliminated. These cells play a critical role in the development of “immune tolerance” to certain foreign antigens, such as those found in food or expressed by commensal (helpful) bacteria in the intestine.

#### B cells

B cells arise from hematopoietic stem cells in the bone marrow and, following maturation, express unique antigen-specific receptors on their membrane. Unlike T cells, B cells can recognize and respond to antigens directly, without the need for APCs, through distinctive antibodies expressed on their cell surface. Each B cell expresses an antibody that recognizes only a single antigen structure, which does not have to be a protein peptide. Antibodies can also recognize lipid and carbohydrate structures produced by pathogens. The principal function of B cells is the production of antibodies against foreign antigens. This requires their further differentiation [3, 5]. Under certain circumstances, B cells can also act as APCs.

B cells undergo proliferation when activated by antigens (e.g., a particular structure on a bacterial cell surface to which they have an appropriate antigen-specific receptor). Usually with the help of signals from CD4 T cells and local cytokines, they differentiate into antibody-secreting plasma cells or memory B cells (see Fig. 2). Memory B cells are “long-lived” survivors of past infection and continue to express antigen-binding receptors. These cells can be called upon to respond quickly by producing antibodies that eliminate an antigen upon re-exposure. Plasma cells, on the other hand, are mostly shorter lived and produce large amounts of antibody that enters the circulation and tissues providing effective protection against pathogens. Given their function in antibody production, B cells play a major role in antibody-mediated immune responses (described below) as opposed to the cell-mediated immune response which is governed primarily by T cells [3, 5].

## Antibody-mediated vs. cell-mediated immunity

Antibody-mediated immunity is the branch of the adaptive immune system mediated by B-cell-antibody production. The antibody-production pathway begins when the B cell’s antigen-binding receptor recognizes and binds to antigen in its native form. Local Th cells secrete cytokines that help the B cell multiply and direct the type of antibody that will be produced. Some cytokines, such as IL-6, help B-cells to mature into antibody-secreting plasma cells. The secreted antibodies bind to antigens on the surface of pathogens, flagging them for destruction through complement activation, opsonin promotion of phagocytosis and pathogen elimination by immune effector cells. Upon elimination of the pathogen, the antigen–antibody complexes are cleared by the complement cascade, while debris and dead cells are cleared by phagocytes (see Fig. 2) [5].

Five major types of antibodies are produced by B cells: IgA, IgD, IgE, IgG, and IgM. IgG antibodies can be further subdivided into structurally distinct subclasses with differing abilities to fix complement, act as opsonins, and neutralize toxins and viruses. The major classes of antibodies have substantially different biological functions and recognize and neutralize specific pathogens. Table 2 summarizes the various functions of the five Ig antibodies [6]. Antibodies play an important role in containing viral infection during the acute phase or in preventing viruses from entering cells. They do this by binding to viral particles and preventing their entry into cells, as well as opsonizing cells that express viral antigens. However, they are not, on their own, generally capable of eliminating a virus once infection has occurred. Once an infection is established, cell-mediated immune mechanisms are usually the most important defense against intracellular pathogens.

Cell-mediated immunity does not involve antibodies, but rather protects an organism through a variety of other mechanisms which depend on T-cell activity [5]. A key cellular immune process is the activation of antigen-specific cytotoxic T cells that can induce apoptosis of cells displaying foreign antigens or derived peptides on their surface, such as virus-infected cells, cells with intracellular bacteria, and cancer cells displaying tumour antigens. T cell and NK cell production of cytokines, such as IFN-γ and IL-17, further mediates the effective immune response by enhancing the development of cytotoxic (CD8 +) T cells. Similar signals from T cells enhance the activation of macrophages and NK cells, enabling them to destroy intracellular pathogens.

Cell-mediated immunity is directed primarily at microbes that survive in phagocytes as well as those that infect non-phagocytic cells and are retained intracellularly. This type of immunity is most effective in eliminating virus-infected cells and cancer cells, but can also participate in defending against fungi, protozoa, and intracellular bacteria. Cell-mediated immunity also plays a major role in transplant rejection.

## Passive vs. active immunization

Acquired immunity is attained through either passive or active immunization. Passive immunization refers to the transfer of *active* humoral immunity, in the form of “ready-made” antibodies, from one individual to another. It can occur naturally via transplacental or breast milk transfer of maternal antibodies, or it can be induced artificially by injecting a recipient with antibodies that are usually manufactured for this purpose and that are targeted to a specific pathogen or toxin. The latter is used when there is a high risk of infection and insufficient time for the body to develop its own immune response, or to reduce the symptoms of chronic or immunosuppressive diseases. Examples include antibodies to respiratory syncytial virus (RSV) which can be given to high-risk infants to prevent RSV infection, or pooled Ig provided to subjects who, for genetic or other reasons, do not mount appropriate antibody responses on their own.

Active immunization refers to the production of antibodies against a specific antigen or pathogen *after* exposure to the antigen. This process, in the form of vaccination, has been highly effective in preventing or reducing the severity of many infectious diseases, including influenza and coronavirus disease (COVID-19). A vaccine can consist of inactivated organisms, specific proteins or carbohydrates known to induce immunity, or messenger RNA for important proteins for the pathogens. Attenuated (weakened) pathogens are also sometimes used for immunization. Through vaccinations, the immune system develops a memory response to the potential pathogen. If it is encountered in the future, the immune system can either combat it completely, so that the individual does not get ill, or it can reduce the severity of infection substantially. Effective active immunization often requires the use of “adjuvants” which improve the ability of the immune system to respond to antigen injection.

Acquired immunity can also be acquired through natural infection with a microbe, and protection from further infection with the same microbe can be life-long.

## Immunopathology

As mentioned earlier, defects or malfunctions in either the innate or adaptive immune response can provoke illness or disease. Such disorders are generally caused by an overactive immune response (known as hypersensitivity reactions), an inappropriate reaction to self (known as autoimmunity) or ineffective immune responses (known as immunodeficiency).

### Hypersensitivity reactions

Hypersensitivity reactions refer to undesirable responses produced by the normal immune system. There are four types of hypersensitivity reactions [7, 8]:

- Type I: immediate hypersensitivity
- Type II: cytotoxic or antibody-dependent hypersensitivity
- Type III: immune complex disease
- Type IV: delayed-type hypersensitivity

A brief summary of these four types of hypersensitivity reactions is provided in Table 3.

**Table 3:** Types of hypersensitivity reactions [7, 8]

Type	Alternate name	Examples	Mediators
I	Allergy (immediate)	• Atopy - Anaphylaxis - Asthma - Allergic rhinitis - Angioedema - Food allergy	IgE
II	Cytotoxic, antibody-dependent	• Erythroblastosis fetalis • Goodpasture syndrome • Autoimmune anemias, thrombocytopenias	IgG, IgM
III	Immune complex disease	• Systemic lupus erythematosus • Serum sickness • Reactive arthritis • Arthus reaction	Aggregation of antigens IgG, IgM Complement proteins
IV	Delayed-type hypersensitivity, cell-mediated, antibody-independent	• Contact dermatitis (e.g. poison ivy) • Tuberculosis skin test • Chronic transplant rejection	T cells, monocytes, macrophages

Type I hypersensitivity is the most common type of hypersensitivity reaction. It is an allergic reaction provoked by re-exposure to a specific type of antigen, referred to as an allergen. Unlike the normal immune response, the type I hypersensitivity response is characterized by the secretion of IgE by plasma cells. IgE antibodies bind to receptors on the surface of tissue mast cells and blood basophils, causing them to be “sensitized”. Later exposure to the same allergen cross-links the bound IgE on sensitized cells resulting in degranulation and the secretion of active mediators, such as histamine, leukotrienes, and prostaglandins, that cause vasodilation and smooth-muscle contraction of the surrounding tissue. Common environmental allergens inducing IgE-mediated allergies include pet (e.g., cat, dog, horse) epithelium, pollen, house dust mites, and molds. Food allergens are also a common cause of type I hypersensitivity reactions (see *IgE-mediated Food Allergy* article in this supplement); however, these reactions are more frequent in children than adults. Treatment of type I reactions generally involves trigger avoidance, and in the case of inhaled allergens, pharmacological intervention with bronchodilators, antihistamines and anti-inflammatory agents. Some types of allergic disease can be treated with immunotherapy (see *Allergen Immunotherapy* article in this supplement). Severe cases of type 1 hypersensitivity, such as anaphylaxis (see *Anaphylaxis* article in this supplement), may require immediate treatment with epinephrine.

Type II hypersensitivity reactions are rare and take anywhere from 2 to 24 h to develop. These types of reactions occur when IgG and IgM antibodies bind to the patient’s own cell-surface molecules, forming complexes that activate the complement system. This, in turn, leads to opsonization, red blood cell agglutination (process of agglutinating or “clumping together” if the antigen is on the surface of red blood cells), cell lysis and death. Some examples of type II hypersensitivity reactions include erythroblastosis fetalis, Goodpasture syndrome, and autoimmune anemias in which autoantibodies bind to red cells on other tissues, such as the lung.

Type III hypersensitivity reactions occur when IgG and IgM antibodies bind to soluble proteins (rather than cell surface molecules as in type II hypersensitivity reactions) forming immune complexes that can deposit in tissues, leading to complement activation, inflammation, neutrophil influx, and mast cell degranulation. This type of reaction can take days, or even weeks, to develop and treatment generally involves anti-inflammatory agents and corticosteroids. Examples of type III hypersensitivity reactions occur in systemic lupus erythematosus, serum sickness, and reactive arthritis.

Unlike the other types of hypersensitivity reactions, type IV reactions are cell-mediated and antibody-independent. They are the second most common type of hypersensitivity reaction, and usually take 2 or more days to develop. Type IV reactions are caused by the overstimulation of T cells and monocytes/macrophages which leads to the release of cytokines that cause inflammation, cell death and tissue damage. In general, these reactions are easily resolvable through trigger avoidance and the use of topical corticosteroids. An example of a type IV reaction is the inflamed skin response to poison ivy.

### Autoimmunity

Autoimmunity involves the loss of normal immune homeostasis such that the organism produces an abnormal response to its own tissue. The hallmark of autoimmunity is the presence of self-reactive T cells, auto-antibodies, and inflammation. Prominent examples of autoimmune diseases include: rheumatoid arthritis, type 1 diabetes mellitus and Graves’ disease [9].

### Inflammation

Defects in immune regulation are associated with many chronic inflammatory diseases, whether they are autoimmune in nature or their causes are less well understood. Poorly regulated inflammatory responses and tissue damage as a result of inflammation are often immunopathological features. Inflammatory diseases include: rheumatoid arthritis, psoriasis, inflammatory bowel disease and chronic asthma. The classical features of inflammation are heat, redness, swelling and pain. Inflammation can be part of the normal host response to infection and a required process to rid the body of pathogens, or it may become uncontrolled, ongoing, and lead to chronic inflammatory disease. The overproduction of inflammatory cytokines (such as TNF, IL-1, and IL-6) as well as the recruitment of inflammatory cells (such as neutrophils and monocytes) through the function of chemokines are important drivers of the inflammatory process. Additional mediators produced by recruited and activated immune cells induce changes in vascular permeability and pain sensitivity. Therapies for inflammation include both agents that block these mediators and, more recently, agents that target the cytokines driving the inflammatory process directly.

### Immunodeficiency

Immunodeficiency refers to a state in which the immune system's ability to function properly to fight infectious disease, or function appropriately in other ways, is substantially compromised. Immunodeficiency disorders may result from a primary genetic defect (primary immunodeficiency [also referred to as inborn errors of immunity [IEI]—see *IEI* article in this supplement) which can effect either innate or acquired immune function through inhibition of selected immune cells or pathways [10], or they may be acquired from a secondary cause (secondary immunodeficiency—see *Secondary Immunodeficiency* article in this supplement), such as viral or bacterial infections, malnutrition, autoimmunity or treatment with drugs that induce immunosuppression, such as certain anti-cancer drugs [11]. Certain diseases can also directly or indirectly impair the immune system such as leukemia and multiple myeloma. Immunodeficiency is also the hallmark of acquired immunodeficiency syndrome (AIDS), caused by the human immunodeficiency virus (HIV). HIV directly infects Th cells and impairs other immune system responses indirectly [11].

## Conclusion

Innate immunity is the first-line immunological, non-pathogen-specific mechanism for fighting against infections. This immune response is rapid, occurring minutes or hours after aggression, and is mediated by many enhanced barrier activities to prevent infection, numerous cells including phagocytes, mast cells, basophils, and eosinophils, as well as the complement system. Adaptive immunity develops in conjunction with, and is enhanced by, innate immunity to eliminate infectious agents and defend against re-infection. It relies on the tightly regulated interplay between T cells, APCs, and B cells. A critical feature of adaptive immunity is the development of immunologic memory or the ability of the system to learn or record its experiences with various pathogens, leading to effective and rapid immune responses upon subsequent exposure to the same or similar pathogens. The adaptive immune response, particularly the production of antibodies and cytokines can, in turn, enhance the function of innate immune processes, such as complement-mediated destruction of pathogens and the ability of phagocytes to kill bacteria. A brief overview of the defining features of innate and adaptive immunity are presented in Table 4.

**Table 4:** Overview of the defining features of innate and adaptive immunity [1]

	Innate immune system	Adaptive immune system
Cells	Hematopoietic cells: • Macrophages • Monocytes • Dendritic cells • Mast cells • Neutrophils • Basophils • Eosinophils • NK cells Non-hematopoietic cells: • Epithelial cells (skin, airways, gastrointestinal tract) • Endothelial cells	Hematopoietic cells: • T cells • B cells Dendritic cells (APCs)
Molecules	• Cytokines • Complement • Proteins and glycoprotein	• Antibodies (Ig) • Cytokines
Response time	• Immediate	• Delayed by hours to days
Immunologic memory	• None: responses are the same with each exposure	• Responsiveness enhanced by repeated antigen exposure

There is a great deal of synergy between the adaptive immune system and its innate counterpart, and defects in either system can lead to immunopathological disorders, including autoimmune diseases, immunodeficiencies and hypersensitivity reactions. The remainder of this supplement will focus on the appropriate diagnosis, treatment and management of some of these more prominent immune-mediated disorders.

## Data Availability

Not applicable.

## Abbreviations

AIDS: Acquired immunodeficiency syndrome
APCs: Antigen-presenting cells
COVID-19: Coronavirus disease 2019
DAMPs: Damage-associated molecular patterns
HIV: Human immunodeficiency virus
HLA: Human leukocyte antigen
IEI: Inborn errors of immunity
IFN- γ: Interferon-gamma
Ig: Immunoglobulin
IL: Interleukin
ILCs: Innate lymphoid cells
LPS: Lipopolysaccharides
MHC: Major histocompatibility complex
NK: Natural killer
PAMPs: Pathogen associated molecular patterns
PRRs: Pattern recognition receptors
RNA: Ribonucleic acid
RSV: Respiratory syncytial virus
SARS-CoV-2: Severe acute respiratory syndrome coronavirus 2
TCR: T-cell receptor
TNF: Tumour necrosis factor
T reg: Regulatory T cell
